# Intervening with Urinary Tract Infections Using Anti-Adhesives Based on the Crystal Structure of the FimH–Oligomannose-3 Complex

**DOI:** 10.1371/journal.pone.0002040

**Published:** 2008-04-30

**Authors:** Adinda Wellens, Corinne Garofalo, Hien Nguyen, Nani Van Gerven, Rikard Slättegård, Jean-Pierre Hernalsteens, Lode Wyns, Stefan Oscarson, Henri De Greve, Scott Hultgren, Julie Bouckaert

**Affiliations:** 1 Department of Molecular and Cellular Interactions, Vrije Universiteit Brussel, Brussels, Belgium; 2 Ultrastructure, Vrije Universiteit Brussel, Brussels, Belgium; 3 Department of Molecular Microbiology, Washington University School of Medicine, St. Louis, Missouri, United States of America; 4 Viral Genetics, Vrije Universiteit Brussel, Brussels, Belgium; 5 Department of Organic Chemistry, Arrhenius Laboratory, Stockholm University, Stockholm, Sweden; 6 Centre for Synthesis and Chemical Biology, University College Dublin, Belfield, Dublin, Ireland; Massachusetts Institute of Technology, United States of America

## Abstract

**Background:**

*Escherichia coli* strains adhere to the normally sterile human uroepithelium using type 1 pili, that are long, hairy surface organelles exposing a mannose-binding FimH adhesin at the tip. A small percentage of adhered bacteria can successfully invade bladder cells, presumably via pathways mediated by the high-mannosylated uroplakin-Ia and α3β1 integrins found throughout the uroepithelium. Invaded bacteria replicate and mature into dense, biofilm-like inclusions in preparation of fluxing and of infection of neighbouring cells, being the major cause of the troublesome recurrent urinary tract infections.

**Methodology/Principal Findings:**

We demonstrate that α-d-mannose based inhibitors of FimH not only block bacterial adhesion on uroepithelial cells but also antagonize invasion and biofilm formation. Heptyl α-d-mannose prevents binding of type 1-piliated *E. coli* to the human bladder cell line 5637 and reduces both adhesion and invasion of the UTI89 cystitis isolate instilled in mouse bladder via catheterization. Heptyl α-d-mannose also specifically inhibited biofilm formation at micromolar concentrations. The structural basis of the great inhibitory potential of alkyl and aryl α-d-mannosides was elucidated in the crystal structure of the FimH receptor-binding domain in complex with oligomannose-3. FimH interacts with Manα1,3Manβ1,4GlcNAcβ1,4GlcNAc in an extended binding site. The interactions along the α1,3 glycosidic bond and the first β1,4 linkage to the chitobiose unit are conserved with those of FimH with butyl α-d-mannose. The strong stacking of the central mannose with the aromatic ring of Tyr48 is congruent with the high affinity found for synthetic inhibitors in which this mannose is substituted for by an aromatic group.

**Conclusions/Significance:**

The potential of ligand-based design of antagonists of urinary tract infections is ruled by the structural mimicry of natural epitopes and extends into blocking of bacterial invasion, intracellular growth and capacity to fluxing and of recurrence of the infection.

## Introduction

Pili and fimbriae on the bacterial cell are virulence factors that mediate adhesion of pathogenic bacteria to host cell receptors [1]. Urinary tract infections (UTIs) in humans are frequently caused by uropathogenic *Escherichia coli* (UPEC) expressing type 1 pili. The FimH adhesin at the tip of type 1 pili recognizes terminal mannose units of uroplakin Ia (UPIa), a membrane glycoprotein that is abundantly expressed on superficial epithelial umbrella cells of the urinary tract [2]. Bacterial attachment stimulates the innate host immune system in a Toll-like receptor 4-dependent manner [3]. This induces the secretion of cytokines by the urothelial cells and recruitment of neutrophils to the mucosal surfaces for the elimination of the bacteria [4]. A subpopulation of UPEC escapes this eradication mechanism of the host by invading into the large superficial epithelial cells in a type 1 pili-dependent mechanism [5], [6]. However, hosts with a robust and timely innate immune response manage to get rid of this bacterial intracellular nesting by exfoliation of the large, superficial umbrella cells and discharge of these infected cells with the urine [7], [8].

Bacteria within the cytosol of umbrella cells replicate and within hours develop into tightly packed, biofilm-like intracellular bacterial communities (IBCs) [9]. Upon maturation of the IBCs, the bacteria disperse from the IBCs and re-emerge in the bladder lumen in long, filamentous shapes that helps them to evade neutrophil phagocytosis [10], [11]. They can then reinvade neighbouring epithelial cells to re-establish infection. As such, even after the acute infection is resolved, bacteria can remain within the bladder for many days to weeks, regardless of standard antibiotic treatments, and can be implicated in recurrent urinary tract infection (rUTI) [12]–[14]. Most UPEC isolates from women with acute or rUTIs, asymptomatic bacteriuria and pyelonepritis replicate in IBCs in C3H/HeN mice, although IBCs from isolates associated with acute UTIs remained significantly smaller [15]. UPEC that are unable to express type 1 pili are dramatically attenuated in their virulence, refrain from intracellular aggregation and maturation into an IBC and therefore fail to flux back out of the cells [16].

A different, intracellular path of the bacteria is commenced through the endocytosis in the fusiform or discoidal vesicles of superficial umbrella cells [17]. The bacteria make use of the vesicle trafficking in the umbrella cells to escape elimination during voiding. Endocytosis in the umbrella cells is coupled to exocytosis of secretory lysosomes [18]. Exocytosis helps to enlarge the apical membrane during bladder filling under hydrostatic pressure. High intracellular cAMP and calcium levels enhance exocytosis of the UPECs back into the lumen of the bladder [17]. It is unclear whether the bacteria are fit enough following their stay in the umbrella cell lysosomes to start another invasive cycle [19]. The uroepithelial cell layer underlying the umbrella cells can also be subject to invading bacteria, possibly upon the incomplete elimination of type 1 piliated *E. coli* during exfoliation of the superficial, highly differentiated umbrella cells [8]. In those immature cells, the bacteria do not reside in the cytosol but rather are sequestered in late endosomes or lysosomes where they remain in a non-replicating state [20]. Those quiescent intracellular reservoirs (QIRs) persist for months even in the face of antibiotics and the host defense, that mainly attack growing bacteria [8], [12], [13]. Only upon differentiation of the immature host cells and rearrangement of the cytoskeleton that tends to inhibit the intracellular replication, the QIRs resolve to develop intracellular inclusions similar to experimental IBCs [20]. Host cytokeratin intermediate filaments are closely associated with and probably help with biofilm formation within these pod-shaped inclusions [20].

The attachment of adhesins on the bacterial cell surface to definite carbohydrates on the host tissue surface is considered to be an initial and critical step in pathogenesis. Antibodies that specifically block the binding of FimH to its natural receptor also prevent infection [21]. Revealing the interaction between FimH and its structural receptor would allow the design of carbohydrate-based anti-adhesives [22]. The preference of the fimbrial adhesin for structures found in mannosylated *N*-linked glycan chains on eukaryotic cell glycoproteins suggested that these structures would serve as the receptors for type 1-piliated *E. coli* strains [23], [24]. The group of Sharon described in the early eighties the strong inhibitory potency of Manα1,3Manβ1,4GlcNAc on the agglutination of red blood cells and yeast cells and suggested that this trisaccharide would best fit the FimH binding pocket [25]. More recently, equilibrium dissociation constants (K_d_) derived from surface plasmon resonance (SPR) solution affinity measurements or from the displacement of tritiated mannose from the receptor-binding domain (RBD) of FimH, consistently approved the strong preference of FimH for oligomannosides exposing Manα1,3Man at the non-reducing end of the D1 arm of high-mannose glycans [26]. Mannotriose and mannopentaose do not exhibit a significantly higher affinity than their linear moiety Manα1,3Man (K_d_∼200 nM). Conversely, oligomannose-3 and oligomannose-5, carrying the β1,4-linked chitobiose as an anchor to an asparagine in the Asn-X-Ser/Thr motif of *N*-linked glycoproteins, have another 10-fold increase in affinity for the FimH RBD (K_d_∼20 nM) over Manα1,3Man [26].

Regardless of the well-characterized fine-specificity of FimH, FimH variant strains have also been categorized into those with a high affinity for d-mannose, predominantly represented by UPECs, *versus* fecal, commensal *E. coli* strains that only displayed an intermediate affinity for mannotriose (Manα1,6(Manα1,3)Man) [27]. These different bacterial binding characteristics under static binding conditions had been attributed to variation in the FimH RBD. Nonetheless, the isolated RBDs of all FimH variants, except for the enterohaemoraghic isolates carrying the Asn135Lys mutation in their pocket, do not differ in their affinities for high-mannose glycans [26], [28]. Isolated RBDs or fimbrial tip adhesin that are out of the context of their own fimbriae have recently been suggested to always occur in a high-affinity conformation [29]. On the other hand, when present within their endogenous pilus, the adhesin can undergo a transition to the high-affinity conformation under shear force enhanced adhesion, and it is this phenomenom where amino acid variation in FimH can give rise to ten-fold differences in bacterial adhesion. Shear enhanced bacterial adhesion was most pronounced to layers coated with the weakly binding mannose than to a surface coated with the more specific mannotriose and involved a minimum of shear for the fecal FimH variant *E. coli* F18 strain [30]. However, no such shear threshold was observed for type 1-piliated *E. coli* binding to mannotriose or for a P-piliated pyelonephritis strain binding to Galα1,4Gal [31]. Thus changes in the receptor structure, that affect the fine-specificity of FimH, can more dramatically than FimH variation impact on the colonization behaviour of type 1-piliated bacteria under flow conditions. In this context, the relevance of a shear threshold observed for single-exposed mannose is feeble given that mannose, recognized by FimH at the termini of naturally occurring glycans, is almost invariably involved in a glycosidic linkage.

FimH has been reported to bind to several glycosylation-dependent receptors in the urinary tract, among which are uroplakin Ia (UPIa) [2], Tamm-Horsfall glycoprotein (THP) [32] and very recently β1 and α3 integrins [33]. Many pathogens gain entry into target host cells by binding integrins either directly or indirectly via the recognition of extracellular matrix proteins. UPIa is an integral membrane protein of the large superficial epithelial cells of the bladder located as a FimH receptor on the six inner domains of the uroepithelial plaque particle [34]. It belongs to the superfamily of tetraspanins [35] that are often found to complex with β1 integrin receptors [36]. Mouse UPIa4 presents high-mannose glycans on Asn169 with a heterogeneity ranging from Man_6_GlcNAc_2_ to Man_9_GlcNAc_2_ [37]. The same high-mannose type glycans decorate β1 and α3 integrins [38]. None of these structures expose Manα1,3Manβ1,4GlcNAc, terminally at the non-reducing end of the glycan branch. Perhaps this is not so striking, as glycoproteins bearing Man_5_GlcNAc_2_ glycans are degraded preferentially [39]. The only isomer of oligomannose-6 encountered so far on uroplakins covers Manα1,3Manβ1,4GlcNAc with an extra α1,2-linked d-mannose [37], thus masking the epitope with high affinity for FimH [40]. THP is secreted in the urine as a natural inhibitor of type 1-mediated bacterial adhesion through its high-mannosylated Asn251 residue [41].

UTIs are one of the most prevalent infections for humans. Almost half of all women will experience at least one UTI in their lifetime. More problematic is the evolution of acute UTIs into chronic infections, with recurrence of the symptoms two or more times within months of a primary infection [42]. Modifications in the glycosylation of FimH receptor proteins on eukaryotic cells may alter the host sensitivity to UTI causing strains. For example, diabetic patients and elderly women show increased bladder cell binding by FimH [43] and this is further correlated with an increased frequency of asymptomatic bacteriuria [44]. Free Manα1,3Manβ1,4GlcNAc oligosaccharide can be isolated in abnormally high amounts from urine of patients with mannosidosis [45]. For mannosidosis patients the abundance of this high-affinity FimH epitope in the urine may act as a natural inhibitor for urinary tract infections, although a decreased risk of UTIs in patients with α-mannosidase deficiency has not been described. The high frequency of recurrent infections and the increasing antibiotic resistances of UPECs [46] highlight the need for alternative treatments using carbohydrate-derived molecules as potential anti-adhesives. Their non-bactericidal effect makes the selection of strains resistant to such agents much more unlikely than those resistant to antibiotics [22].

Biochemical and docking studies predicted that the enhanced binding of Manα1,3Manβ1,4GlcNAc is accomplished by interactions of the central mannose and GlcNAc in the tyrosine gate extending from the FimH mannose-binding pocket [26], [47]. To gain insight into the selectivity of FimH for this trisaccharide epitope, we co-crystallized FimH with the trisaccharide-presenting oligomannose-3 and investigated the importance of the glycosidic linkage of Manα1,3Man with *N*-acetyl glucosamine (GlcNAc) of the chitobiose unit of *N*-linked glycans. The FimH RBD shows high affinities for alkyl α-d-mannosides, with affinities up to a K_d_ of 5 nM for heptyl α-d-mannose (HM) [47]. We set out to explore the inhibitory capacity of HM for lower UTIs caused by type 1-piliated UPECs in mice. We find that alkyl α-d-mannosides and other synthetic inhibitors of FimH confirm binding properties of the high-mannose epitope. The alkyl chain of butyl α-d-mannose, bound to FimH in two previously determined co-crystal structures, follows the trail of the α1,3 linkage to the central mannose and the first β1,4 glycosidic linkage to the chitobiose. The potential of ligand-based design of antagonists of UTIs appears to be ruled by structural mimicry of specific spots on mannosylated receptors. This anti-adhesive ability extends into blocking of bacterial invasion, intracellular growth and capacity to fluxing and recurrence of the infection.

## Results

### The chitobiose anchor to Asn-glycosylated FimH receptors is important for specificity

**Figure 1:**
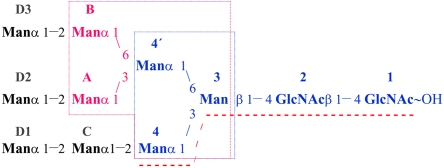
Oligomannose-3 as part of the high-mannose glycans. Oligomannose-9 (K_d_∼420 nM) is the full structure here displayed. Oligomannose-3 (blue, K_d_∼20 nM) is the substructure that was crystallized in complex with the FimH adhesin. It interacts with FimH as an extended tetrasaccharide, underlined with a red hatched line.Oligomannose-5 (K_d_∼12 nM) includes an extra mannotriose core over oligomannose-3 through addition of ManA and ManB (fuchsia). Mannopentaose (K_d_∼120 nM) and mannotriose (K_d_∼200 nM) are boxed in fuchsia and blue frames respectively.

Previous epitope mapping on high-mannose glycan receptors revealed the highest affinity of the FimH RBD for oligomannose-3 and oligomannose-5 [26]. Both these oligomannosides expose Manα1,3Manβ1,4GlcNAc at the non-reducing end of the D1 branch and have an increased affinity for FimH over mannotriose and mannopentaose that lack the chitobiose unit (Figure 1). The increased affinity was thought to be due partially to the β-anomeric linkage to the chitobiose GlcNAcβ1,4GlcNAc. The binding constants of the FimH RBD from the J96 cystitis strain for Manα1,3ManβOMe and for the anomeric mixture of Manα1,3Man have been determined using SPR, to investigate the contribution of the glycosidic linkage. This resulted in affinities of K_d_ = 112 nM and 281 nM respectively, showing that FimH selects out the β-anomeric configuration on Man3. It also indicated that the presence of the β-linkage alone is not sufficient to explain the more significant increase in affinity between on the one hand mannotriose or mannopentaose, and on the other hand oligomannose-3 and oligomannose-5 [26]. Thus the chitobiose unit that bridges the mannosides to the asparagine in the Asn-X-Ser/Thr motif of the glycoprotein receptor contributes more significantly to the interaction with FimH.

### Crystal structure of the FimH receptor-binding domain in complex with oligomannose-3

Oligomannose-3 has been crystallized in complex with the FimH RBD. This oligosaccharide exposes the substructure Manα1,3Manβ1,4GlcNAc terminally on its D1 branch (Figure 1). Therefore it is ideally suited to reveal the structural basis of its high-affinity interaction with FimH. Initial crystals have been grown by equilibration through vapor diffusion and the crystallization condition was optimized through the addition of 3% glycerol (Figure S1) .

**Figure 2:**
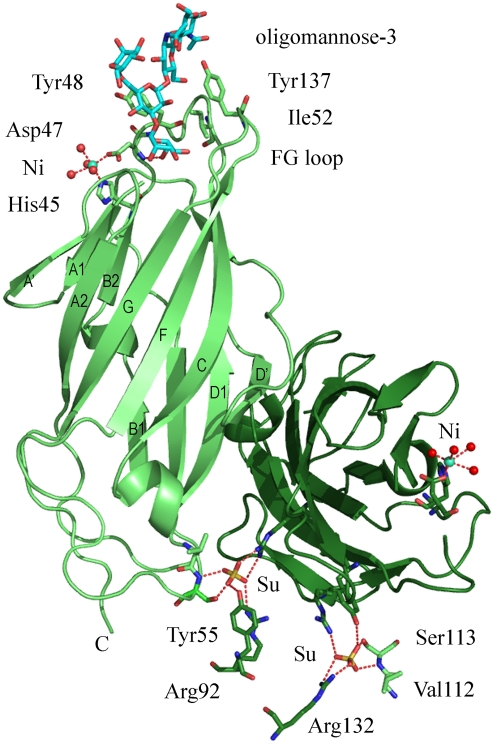
Crystal structure of FimH in complex with oligomannose-3. The two FimH RBDs related by non-crystallographic symmetry (green and forest-green, Ig-fold labeling) both bind oligomannose-3 (only one is shown in blue cyan), interconnected via Asp47 to a nickel ion (green cyan). His45 and Asp47 and four waters ligate the nickel ion in an octahedral configuration. Sulphate ions (yellow with red oxygens) stabilize a highly charged crystal packing interface by bridging Val112 and Ser113 with Arg132 from the two crystallographically independent RBDs with Arg92 and Tyr55 from a symmetry-related RBD.

The solution of the molecular replacement contains two FimH RBDs per asymmetric unit in the P3_1_21 space group (Figure 2, Table S1). Upon the first atomic refinement a clearly interpretable electron density for Manα1,3Manβ1,4GlcNAc was visible, equally well in both RBDs. Refinement against the crystallographic data with a 2.1 Å high-resolution cut-off led to good protein geometry and oligosaccharide conformation determination. The root mean square deviation between the two RBDs is 0.56 Å for all main chain atoms. The torsion angles of the Manβ1,4GlcNAc and Manα1,3Man glycosidic bonds in oligomannose-3 resemble within a few degrees to those observed in the crystal structure of the Manα1,3Manβ1,4GlcNAc trisaccharide [48]. Although the ψ torsion angle of Manα1,3Man deviates from the average minimum energy modelled for these glycans for all distinct conformers of glycosidic linkages found in either *N*- or *O*-linked glycans [49], it falls well within the range of allowed minimum energy conformations for φ and ψ glycosidic torsion angles [50].

**Figure 3:**
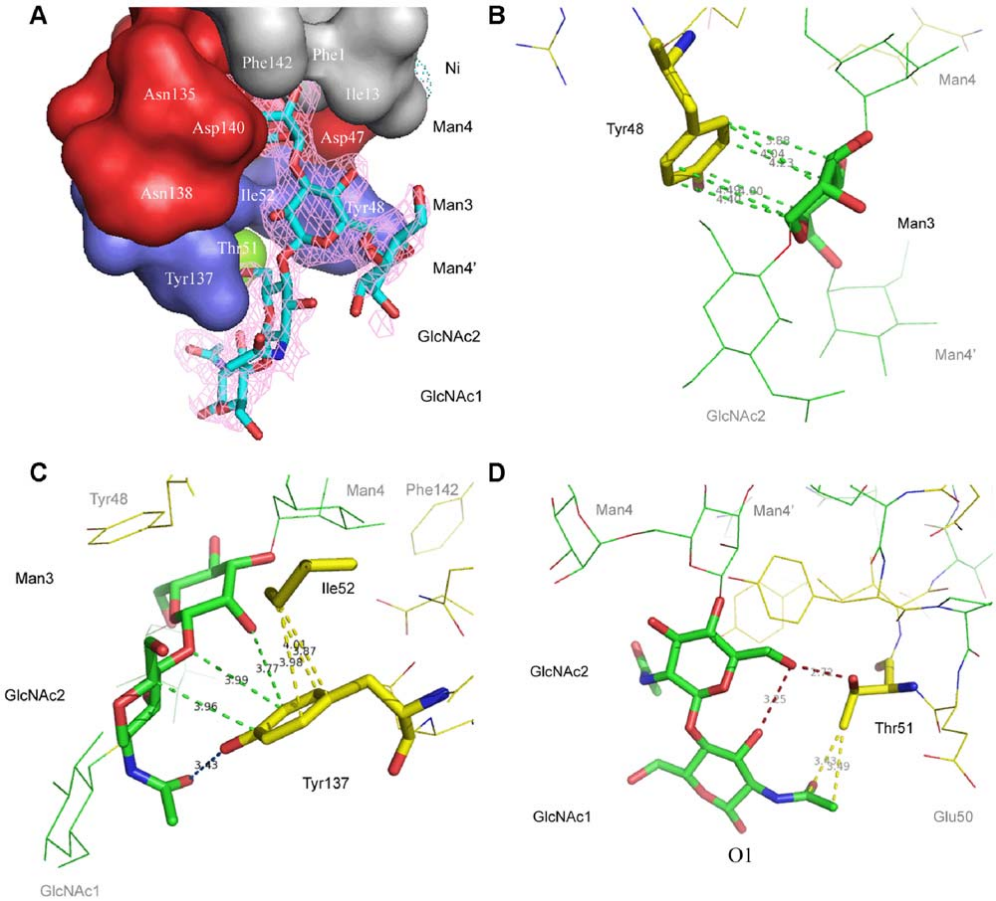
Panel of FimH lectin domain interactions along the oligomannose-3 chain. A, Electron density map, contoured at 1 e/Å^3^, for oligomannose-3 in the FimH receptor-binding site. The surface of the binding site is subdivided into its hydrophobic support platform (grey, residues Phe142, Phe1 and Ile13), its polar pocket (red, residues Asn46, Asp47, Asp54, Gln133, Asn135, Asn138 and Asp140), the tyrosine gate (blue, residues Tyr137, Ile52 and Tyr48) and residue Thr51 (green). B, Aromatic-to-saccharide stacking (green dashed lines) of the Tyr48 side chain onto the B-face of Man3. C, van der Waals support of the β1,4 linkage (yellow dashed lines) and an hydrophobic contact of C5 of GlcNAc2 by Tyr137 (green dashed lines). D, Thr51 tops of the site by hydrophilic (red), hydrophobic and van der Waals (yellow) interactions with the chitobiose. The anomeric O1 of GlcNAc1 would be exchanged for by the nitrogen of the amide of aspargine on a receptor glycoprotein for FimH.

Oligomannose-3 conforms into a relatively planar structure that inserts almost like a sheet into the tyrosine gate (Figures 2 and 3A), with an angle at both ends. The same extensive hydrogen bonding, hydrophobic contacts and van der Waals interactions are achieved by binding of oligomannose-3 in both FimH RBDs of the crystal structure (Figure 2). The solvent accessible surface area buried through the binding of oligomannose-3 leads to a reduction of solvent accessible surface area of 312.1±1.3 Å^2^ for oligomannose-3 and 462.2±6,6 Å^2^ for FimH both, using the CCP4 program NACCESS. Between the two crystallographically-independent FimH RBDs 537.5±6.75 Å^2^ is excluded from the solvent. This agrees well with the average areas excluded from the solvent by pairwise interactions (388.1±3 Å^2^ between FimH and oligomannose-3 and 534 Å^2^ between the two FimH RBDs) calculated by MSDpisa version v1.14 [51].

The non-reducing end Man4 anchors into the mannose-binding pocket, whereas the reducing end GlcNAc1 folds over Thr51. Very specific interactions with the tyrosine gate occur in the mannose-binding pocket itself and all along Manα1,3Manβ1,4GlcNAc via the α1,3 and the first β1,4 glycosidic linkages. FimH is not directly recognizing the second non-reducing end mannose, Man4' (Figure 3). Man4' and GlcNAc1, the two saccharides that exit from the receptor-binding site, display a larger flexibility in an extensively hydrated environment, resulting in less-well defined electron density and higher temperature factors. Crystal packing contacts with Phe142 and Ile13 of a symmetry-related RBD with Man4' bound to the second RBD (forest-green molecule in Figure 2) stabilize some of these waters, whereas Man4' bound to the first RBD is fully solvent exposed.

For the first time in a FimH structure [47], [52], [53], a nickel ion was observed, interlinked with the mannose binding pocket by Asp47 (Figure 2). The nickel ion is ligated in an octahedral setting by the carboxylate group of Asp47, the imidazole group of His45 and four water molecules. Also unseen before in any FimH structure are two sulphate ions in a highly charged interface in the crystal packing (Figure 2).

**Figure 4:**
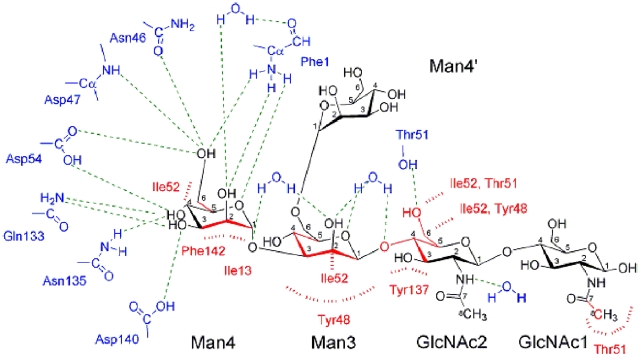
Scheme of the interactions in the extended FimH receptor-binding pocket. A network of hydrogen bonds (green dashed lines) surrounds Man4. Further along oligomannose-3, only water molecules make hydrogen bonds along one side of oligomannose-3 except for the Thr51 side chain. The residues of the tyrosine gate, Tyr48, Ile52 and Tyr137, interact via aromatic stackings, hydrophobic and van der Waals contacts (all marked in red) mainly with Man3 and GlcNAc2.

The non-reducing end Man4 of oligomannose-3 (Figure 1) is attracted to the deep, monomannose-binding polar pocket (Figure 3A, red) through a hydrophobic tyrosine gate (Figure 3A, blue) to make previously well-defined interactions [47], [53]. Eleven hydrogen bonds are formed between the Man4 and the FimH residues Phe1, Asn46, Asp47, Asp54, Gln133, Asn135 and Asp140 (Figure 4). A water molecule interacting with O2 of Man4 is a strongly conserved feature in the mannose binding pocket. The apolar B-face of Man4 provides interaction with Ile13 and Phe142, and its C5-C6 bond interacts with Ile52. Residues Tyr48, Ile52 and Tyr137 of the tyrosine gate are involved in several aromatic/hydrophobic and van der Waals contacts with oligomannose-3, thus stabilizing the glycan-lectin complex (Figures 3 and 4). Man3 hooks over the side chain of Ile52 while stacking its apolar B-face onto the Tyr48 aromatic side chain (Figures 3A–C). A close apolar contact is achieved between C5 of GlcNAc2 and Tyr137. The C6-O6 bond of GlcNAc2 fares well in a hydrophobic environment created by Tyr48 and Ile52 (Figure 4). This could help to orient the strong (2.7 Å) hydrogen bond of the O6 hydroxyl on GlcNAc2 directly towards the hydroxyl of the Thr51 side chain (Figure 3D). This latter threonine interacts hydrophobically with the C7–C8 bond of the acetyl group of the reducing end GlcNAc1. GlcNAc1 intramolecular hydrogen bonds with GlcNAc2 and its *N*-acetyl group makes a van der Waals contact with the methyl group of the Thr51 side chain (Figure 3D). It can be seen that glycosidic linkage of GlcNAc1 via O1 to a glycoprotein would pull FimH very close to its receptor upon binding (Figure 3D).

### Parallels between the binding of oligomannose-3 and alkyl α-d-mannosides with FimH

**Figure 5:**
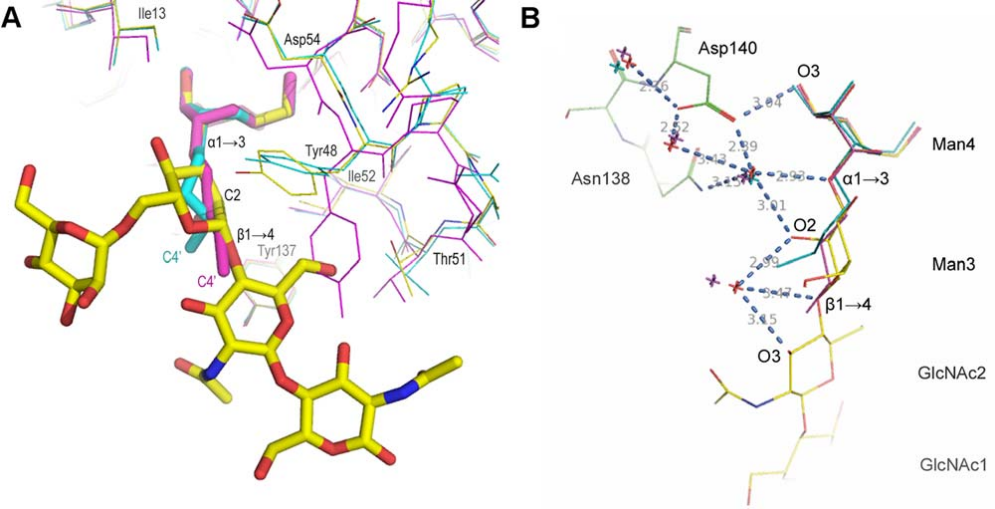
Specificity spots for interaction with FimH. A, Superposing the FimH RBDs with the oligomannose-3 ligand (yellow, PDB entry 2vco, 2.1 Å resolution), with butyl α-d-mannose (magenta, 1uwf, 1.7 Å resolution) and with butyl α-d-mannose (cyan, 1tr7, 2.1 Å resolution). Tyr48 in the 1uwf structure (magenta) adopts a different side chain conformation, for the reason to avoid clashes in the crystal packing. The butyl tail takes on a conformation that matches the C3-C2-C1 greasy trace of the central Man3 almost precisely. B, Water positions in the crystal structures of the FimH RBD (green) in complex with the oligomannose-3 ligand (yellow, waters in red and hydrogen bonds in blue dashed lines) match those of the FimH RBDs of the butyl α-d-mannose-liganded structures (waters in magenta for 1uwf, waters in cyan for 1tr7).

Butyl α-d-mannose is a high-affinity ligand for FimH (K_d_ = 150 nM) that was found serendipitously in two previous FimH crystal structures [47]. We compared the interaction interfaces between FimH and oligomannose-3 with those between FimH and butyl α-d-mannose. Superposing the mannose of the butyl α-d-mannosides onto Man4 of oligomannose-3 and application of the transformation matrix to the whole molecules showed that the butyl chain follows the hydrophobic trail through the tyrosine gate by the Manα1,3Man and Manβ1,4GlcNAc linkages (Figure 5A). In contrast to the stable position of the mannose bound in the monosaccharide-binding pocket, the temperature factors of the ligand atoms gradually increase beyond the α1,3 position, both for oligomannose-3 and for butyl α-d-mannose.

**Figure 6:**
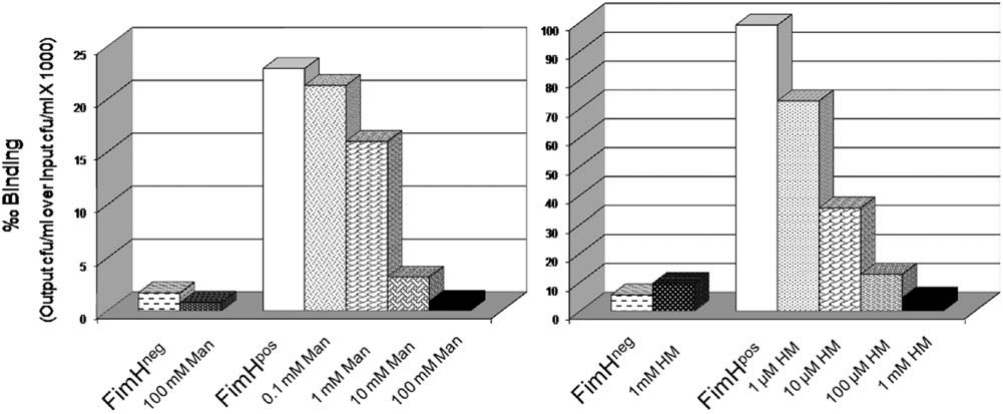
Inhibition of bacterial adhesion to 5637 bladder cells by mannose or heptyl α-d-mannose. Type 1 pili-expressing *E. coli* were incubated at 10^6^ cfu/ml (FimH^pos^) with the bladder cells, or had been mixed with a ten-fold dilution series of Man or HM prior to incubation, to compare inhibition of bacterial binding by the two sugars. An isogenic *fimH*-negative strain served as a negative control (FimH^neg^).

The side of oligomannose-3 that is not embraced by the tyrosine gate displays hydration of the exocyclic glycosidic oxygens (Figures 4 and 5A) that is maintained between oligomannose-3 and butyl α-d-mannose. The hydration on the α-anomeric oxygen of the mannose bound into the polar pocket is conserved, as well as its hydrogen bonding network with Asn138 and Asp140 (Figure 5B) that are residues in the loop connecting β-strands F and G (Figure 3). Also a water molecule hydrogen bonding to the exocyclic glycosidic oxygen linking Man3 to GlcNAc2 is maintained (Figures 5 and 6B). It appears that synthetic inhibitors strive for the best mimic of the interactions of FimH with its natural receptor. Previous solution affinity measurements indicated that heptyl α-d-mannose is the best-binding alkyl α-d-mannoside (K_d_ = 5 nM) [47]. A simulation in which the butyl chain of butyl α-d-mannose was elongated to a heptyl allowed a conformation in which the elongated tail follows GlcNAc2 further along C4-C5-C6, expecting to form the important interactions with Thr51, Ile52 and Tyr48. The anti-adhesive proficiency with heptyl α-d-mannose was subsequently tested both on the human bladder cell line 5637 and in C3H/HeN mice.

### Mannose and heptyl α-d-mannose inhibit *in vitro* adhesion of type 1-piliated *E. coli*

Type 1 pili-expressing AAEC185(pUT2002)(pMMB66) *E. coli* cells at 10^6^ cfu/ml were supplemented with a ten-fold dilution series of d-mannose (Man) or heptyl α-d-mannose (HM) and incubated for adhesion on bladder cell line 5637. Increasing concentrations of Man or HM caused a significant reduction to complete inhibition of bacterial bladder cell binding (Figure 6). Adhesion could be completely inhibited by the addition of 100 mM Man to the bacterial inoculum, or by a 100-fold lower concentration of HM. No inhibition was obtained with 100 µM Man, whereas 1 µM of HM still had some inhibitory effect. Similar data have been obtained with an inoculum of 10^7^ cfu/ml. These results suggest that HM is more efficient at reducing FimH-mediated bacterial adherence, in accordance with its higher affinity for FimH.

### Heptyl α-d-mannose reduces bacterial levels in a murine cystitis model

**Figure 7:**
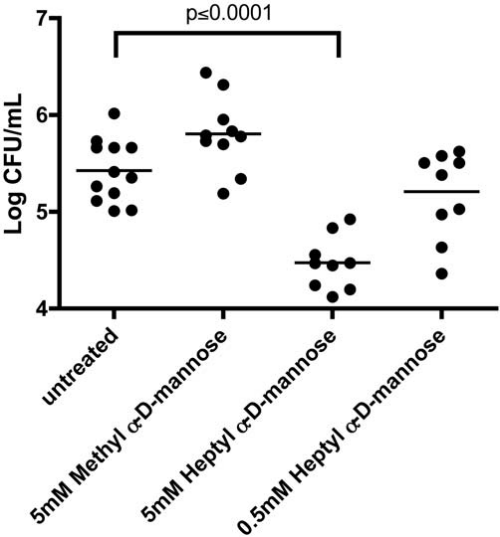
Bacterial load in the mouse bladder 6 hours post-infection. Mice were inoculated with UTI89, either untreated or treated with MM or HM. At 6 hours post-infection there was a significant decrease in the amount of bacteria treated with 5 mM HM in the bladder (p≤0.0001 by Mann Whitney test). This same decrease was not observed with bacteria treated with 5 mM MM. Bacteria treated with 0.5 mM HM were not significantly reduced relative to the untreated infection.

Extensive research has shown that type 1 pili and their adhesin, FimH, are essential for binding and invasion in the murine cystitis model [6], [33], [53]. It was hypothesized that blocking of the FimH binding pocket by preferential binding to a soluble mannose residue would reduce the binding to uroplakins on the epithelial cell surface of the bladder. This loss of binding would result in less invasion and ultimately a reduced infection. To test this hypothesis, bacteria were incubated in the presence of a derivative of mannose prior to inoculation into mouse bladders. Introductory to this experiment, the intrinsic toxicity of HM was assessed in Female BALB/c mice. HM was administered at 50 mM concentration through a catheter (50 µl), intranasal (20 µl) and intravenously (150 µl) to three mice each, but no acute toxicity has been observed. At 6 hours post-infection, the bacteria present within the bladder were enumerated. Wild-type UTI89 incubated with PBS alone had a mean level of infection of ∼3×10^5^ cfu/mL (Figure 7). Incubation of UTI89 with 0.5 mM of HM showed no significant decrease in infection. However, at a concentration of 5 mM HM, there was a significant decrease in infection at 6 hours post-infection (p≤0.0001). Methyl α-d-mannose (MM) gave no decrease in bacterial numbers at the same concentration. A significant decrease in bacterial burden was only observed when increasing the concentration of MM to 1M (data not shown), a 200 fold higher concentration then the one sufficient for HM. The tighter binding of HM to FimH, as compared to MM, could explain this difference. These results suggest that by inhibiting the ability of FimH to bind to mannosylated uroplakins, the bacterial infection can be prevented.

### Heptyl α-d-mannose reduces intracellular bacterial levels

**Figure 8:**
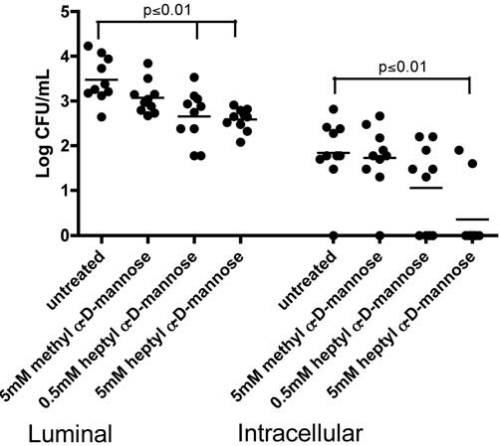
Gentamicin protection assay to determine luminal versus intracellular bacterial population. Mice were inoculated with UTI89 either untreated or treated with MM HM. At 1 hour post-infection, bladders were harvested and processed. Luminal bacteria were obtained from washes of the bladder. The remaining extracellular bacteria were killed with gentamicin and invaded, intracellular bacteria were counted.

Since there is a reduction in the amount of bacteria present in the bladder at 6 hours post-infection with HM incubation (Figure 7), we wanted to assess the amount of bacteria that went intracellular. Mice were infected with UTI89 in PBS or in a MM or HM solution. At 1 hour post-infection, the luminal and intracellular bacterial loads were assessed. There was a significant decrease in the number of luminal bacteria bound to the uroepithelium when treated with 5 mM and 0.5 mM HM (p≤0.01) (Figure 8). This same decrease was not seen when using 5 mM MM. Relative to untreated UTI89, UTI89 incubated with 5 mM HM had a significant decrease in intracellular population (p≤0.01). There was no significant decrease in invaded bacteria treated with 5 mM MM or 0.5 mM HM. These data indicate that HM is interfering not only with type 1 pili-mediated bacterial binding but also with type 1 pili-dependent invasion of UPEC.

### Heptyl α-d-mannose inhibits biofilm formation *in vitro*

**Figure 9:**
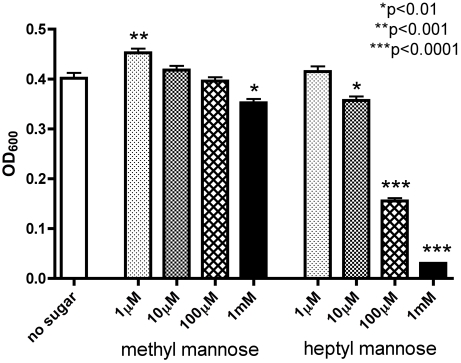
Biofilm formation in the presence of methyl or heptyl α-d-mannose. Standard biofilm assays showed a great reduction of UTI89 biofilm formation in the presence of 100 µM and 1 mM HM (p<0.0001) and a significant increase (p<0.01) in the presence of 1 mM MM.

The development and maturation of IBCs into communities with biofilm-like properties has been shown recently to depend on type 1 pili [16]. We speculated that blocking the FimH binding pocket on type 1 pili would inhibit biofilm formation by inhibiting the adhesive properties of the pili. Bacterial biofilms were grown in the presence of varying concentrations of either HM or MM to elucidate their biofilm inhibiting properties (Figure 9). Biofilm of UTI89 *E. coli* was significantly reduced (p<0.01) only at the highest concentration of MM, 1 mM. However, in the presence of HM, there was a significant decrease in biofilm formation at 10 µM (p<0.001) and both 1 mM and 100 µM concentrations (p<0.0001). This suggests that blocking of the type 1 pili FimH adhesion with HM inhibits biofilm formation. Interestingly, at the lowest concentration of HM (1 µM) there was a significant increase in biofilm formation (p<0.001). A low concentration of a weak inhibitor appears to allow the bacteria to organize into a better matrix through their type 1 pili [54].

## Discussion

### Adhesion, invasion and the formation of intracellular biofilms

UPEC express type 1 pili which bind to mannosylated residues on the surface of epithelial bladder cells. Once bound, some bacteria can invade the cells and rapidly replicate into intracellular bacterial communities, named IBCs, which mature into tightly packed biofilm-like societies [15], [16]. As the infection proceeds, the bacteria filament and flux out of the cells [7], [11]. They can then reinvade neighbouring cells to re-establish infection [14]. Inhibition of UPEC binding to bladder cells will potentially inhibit invasion and limit the bacteria's ability to build up formidable numbers within the bladder. A competitive sugar could potentially disrupt the IBC pathway.

Blocking bacterial invasion could reduce the infection and decrease recurrences. It had been demonstrated already from very early on by Sharon's group that mannose has an inhibitory can inhibit FimH-mediated bacterial adhesion [55]. We looked into the ability of heptyl α-d-mannose (HM) to reduce infection, because HM has an optimized alkyl length for interaction with FimH [47]. Inhibition by soluble mannose (Man) or methyl α-d-mannose (MM) or HM of bacterial adhesion to the bladder cel line *in vitro* or upon transurethral catheterization into the mouse bladder indicates that the bacteria cannot switch between HM and the mannosylated uroplakins. Once HM is bound to FimH on the type 1 pili, the bacteria are irreversibly inhibited in their binding to the bladder surface and the infection is reduced. However, the weak inhibitor MM has a relatively high efficiency in the bladder binding assay that is not in direct correlation with its affinity compared to HM. The affinity of MM for FimH is 500-times less than this of HM, but a concentration of MM a 100-fold higher than the inhibitory concentration of HM is sufficient. The explanation may be in the fact that the *in vitro* adhesion assay is being performed under shaking conditions. Nilsson studied the inhibitory effect of MM on binding of type 1-piliated *E. coli* to mannosylated surfaces under different shear stress conditions. During rolling, FimH molecules transiently bind and detach from surface receptors, allowing a weak soluble inhibitor such as MM to eventually bind to all FimH molecules, thereby preventing further adhesion [54], [56]. In contrast, stationary adhesion is mediated by long-lived adhesin-receptor bonds that prevent inhibitor binding during the time course of the experiments (∼10 min). HM forms such high-affinity intractions with FimH, also under still conditions. The data on the inhibition by MM or HM of adhesion in the C3H/HeN mice indeed pledge that, for a free small-molecule ligand to be an efficient FimH anti-adhesive even under conditions of fluid flow, such as in the bladder, it must bind significantly tighter to FimH than the binding of FimH to the receptor molecules on the bladder cells, in order to overcome the shear-enhanced affinity. HM corresponds to these requirements, having an affinity for FimH higher than any known natural mannosylated receptor. In this context it is interesting to note that whereas human uroplakin UP1a and integrins only carry mannose receptors with intermediate affinity for FimH, the abundantly secreted urinary glycoprotein human THP displays 8% of the high-affinity oligomannose-5 receptors and 75% of oligomannose-6 exposing Manα1,3Man on the D2 branch (Figure 1) [57].

IBCs are biofilm-like groups of bacteria that form upon adhesion, invasion and replication. Biofilm development requires tight interactions between individual cells in the community. Biofilm formation and maturation in UPEC IBCs is type 1-pili dependent [16] and the homotypic interactions between *E. coli* by means of type 1 pili are glycan dependent [58]. Therefore, the ability to disrupt biofilm formation using MM or HM was evaluated. A significant decrease in biofilm formation was observed with as little as 10 µM HM, a 100-fold lower concentration than was needed with MM. The only other data that exist on the inhibition of biofilm formation by a glycan are the inhibition of Gram-positive and Gram-negative biofilms by a secreted group II capsule polysaccharide [59]. The concentration-dependent inhibition of biofilm formation by mannose glycans indicates that the FimH adhesin is involved in biofilm formation [58]. Another very interesting observation is the significant increase in biofilm with very low concentrations of MM (Figure 9). The presence of low concentrations of a soluble inhibitor such as MM during a longer period of time (hours) under conditions of low shear can sometimes help the bacteria to spread further and thus colonize larger surfaces [54]. The absence of shear and the presence of MM loosen the interactions between the adhering bacteria and the surface, allowing them to (re-)organize the biofilm. The extent to which the factors that are important for biofilm formation on abiotic surfaces (our assay) are also applicable for biofilm formation on biotic surfaces (e.g. in IBCs) needs to be further studied. However the observation of bacterial biofilm formation on abiotic surfaces is highly relevant in view of the use of medical implants such as urinary catheters. The glycan-based inhibition of UPEC biofilms is interesting in view of the thought that glycoproteins abundantly secreted in the urine, such as THP, could coat the catheter walls and serve as a glue for type 1-piliated *E. coli* to initiate biofilm growth.

The IBCs, or the non-replicating bacteria in quiescent intracellular reservoirs (QIRs), form a repository that can seed a recurrent infection [12]. It was investigated whether the inhibition of binding and biofilm formation *in vitro* could also reduce this intracellular population during UTIs in a mouse model and thus the likelihood of a recurrence. In the presence of 5 mM HM a significant reduction in bacterial numbers in the bladders occurred. Two-hundred times more MM was needed to obtain the same effect. The results on inhibition with high concentrations of mannose were less consistent, perhaps because FimH can still switch between the mannose on the uroplakins and the mannose in the solution. If this switch happens in time, the bacteria are not urinated out. The adhesin is more tightly bound to HM, thus the bacteria are urinated out before being able to bind to the uroplakins. The tighter binding of the heptyl sugar is potentially reducing the number of UPEC able to bind and invade the bladder cells, and establish a robust infection. To confirm this, an *ex vivo* gentamicin protection assay was performed. This allows enumeration of both the extracellularly bound and intracellular bacteria. There was a significant reduction in both bound and invaded bacteria in the presence of 5 mM HM. In summary, HM is interfering not only with binding but also with invasion of UPEC. A 5 mM concentration of HM is needed for full inhibition of bacterial adhesion *in vivo* (Figures 7 and 8), *versus* a 1 mM concentration *in vitro* (Figure 6). In the mouse model, there is likely some loss of the sugar due to voiding or possibly degradation in the bladder. It is also not clear how the number and nature of mannose receptors in the mouse bladder relate to those on the human bladder cell line 5637. Moreover in the mouse the innate immune responses has an influence on bacterial adhesion and invasion.

What is clear is that the UTI89 cystitis strain binds HM, which deters binding to the bladder cells, limiting invasion, and reducing the level of infection. These data confirm that invasion in bladder cells is type 1-dependent [6], as was demonstrated by the non-invasive character of FimH mannose-binding pocket mutants [53]. Several possible invasion pathways for UPECs have been described. Direct binding of FimH to α3 and β1 integrins has been very recently observed and has been shown to depend upon the glycosylation of the integrins, because treatment of α3/β1 integrins with PNGaseF or EndoHf abolished all binding [33]. Bacterial invasion through glycan-mediated interactions of FimH with integrins could also happen indirectly via cellular receptors of differentiated bladder cells, such as CD151, CD46 and UPIa [36].

### Structure, specificity and drug design

The type 1 pilus fimbrial adhesin FimH was co-crystallized with oligomannose-3 to study in detail the interactions with this most specific and natural glycan receptor for FimH. As expected, the tyrosine gate plays a dominant role in the strength and specificity of these interactions. A remarkable feature when analysing the PHI/PSI-chology of the FimH RBD main chain are the tyrosine residues of the tyrosine gate. Both Tyr48 and Tyr137 and even Ile13 are on the borderline for allowed main chain conformations of the Ramachandran plot [60]. The higher affinity of mannopentaose over mannotriose is not reflected in the oligomannose-5 versus oligomannose-3 affinities [26]. The lack of an increased affinity of oligomannose-5 over oligomannose-3 indicates that the choice of FimH is in both cases directed straightforwardly towards the Manα1,3Manβ1,4GlcNAcβ1,4GlcNAc epitope at the non-reducing end of the D1 arm in both oligosaccharides (Figure 1). Further substitution on the α1,6 linked mannose (Man4') would at least not hinder binding of oligomannose-5 via the same epitope, as Man4' does not interact with FimH and extends into the solvent.

The similarity of interactions found in the crystal structures of FimH with oligomannose-3 and butyl α-d-mannose is remarkably high. The butyl chain follows the hydrophobic trail through the tyrosine gate along the α1,3 and the first β1,4 glycosidic linkage to GlcNAc2 (Figure 5A). The same trail is conservedly hydrated near the presumable α1,3 and the first β1,4 exocyclic glycosidic linkages (Figure 5B). Finally and as expected, the extensive hydrogen network around the non-reducing mannose residue in the mannose-binding pocket of FimH is fully conserved [45].

Considerable interest exists in the molecular basis for FimH-mediated adhesion, being fed by long-standing observations that blocking the FimH-receptor interaction prevents bacterial infection [21], [55]. Structural insight in the basis for specificity into the extended binding site of FimH was lacking until now, seriously hampering and invalidating structure-based design of anti-adhesive molecules using organic chemistry. Due to the great need for anti-adhesives to treat and prevent UTIs, the search was nevertheless continued. Dendrimers of mannose-based inhibitors were most often unable to act multivalently on *E. coli*, however the monovalent moiety of the best inhibitors among those dendrimers contain structures whose interactions can closely mimic those of oligomannose-3, such as glycocluster 4 in the recent study by Touaibia [61]. In these inhibitors, the α-anomeric oxygen of the non-reducing mannose is linked to a phenyl ring, containing an ethyn group in the para position and finalizing in a hydroxyl or ether function. A cocrystal structure for such a complex is unfortunately unknown, however it can be easily imagined that the mannose would anchor in the FimH polar pocket, the α-linkage to the phenyl group would resemble the Manα1,3Man linkage, and the phenyl group would ideally stack with the aromatic side chain of Tyr48. The phenyl to ethyn coupling would strengthen the van der Waals contacts that the Manβ1,4GlcNAc glycosidic linkage makes with Tyr137 (Figure 4). The triple-bond character of the ethyn could replace the stacking between the C4-C5 bond of GlcNAc2 and the aromatic ring of Tyr137 (Figure 4). Finally, the methoxy or methanol carbon atom would mimic the interaction of C6 of GlcNAc2 with Tyr48 and Ile52, and the terminal hydroxyl or ether oxygen may form a hydrogen bond with Thr51 (Figures 3D and 4).

In conclusion, subnanomolar inhibitors precisely mimic the kind of interactions FimH makes with oligomannose-3, and do so in an enhanced fashion dependent on the chemical nature of the synthetic moieties engineered to replace all but the mannoside that enters the monosaccharide binding pocket. The knowledge of all specific interactions between FimH and its natural high-mannose receptor and the possibility to relate the specificity- and affinity determining spots on the ligand with the efficiency of synthetic inhibitors can greatly enhance structure-based drug design against lower UTIs.

## Materials and Methods

### Sugar compounds

Oligomannose-3 was custom-synthesized at the Zelinsky Institute of Organic Chemistry (Russia). Methyl α-d-mannose and d-mannose were purchased from Sigma-Aldrich. Manα1,3ManβOMe (S. Oscarson, to be communicated) and HM [47] have been synthesized. Affinity measurements using surface plasmon resonance (SPR) were performed as described [26]. Briefly, the equilibrium dissociation constants of mannose derivatives for FimH were determined using competition in solution for FimH binding of the sugars with an immobilized Fab fragment from the monoclonal antibody 1C10.

### Purification of FimH

The receptor-binding domain (RBD) of the FimH protein (residues 1 till 158 of UPEC J96) was expressed from plasmid pMMB91 transformed into *E. coli* C43 (DE3) cells. C43 (DE3) (pMMB91) *E. coli* cells were grown in minimal medium containing 40 µg/ml of all the amino acids, 0.4% glucose, 2 µg/ml biotin, 2 µg/ml thiamine, 2 mM MgCl_2_ and 25 µg/ml kanamycin at 37°C. At OD_600nm_ = 0,6 the bacteria cells were induced with 1mM IPTG. After overnight incubation at 37°C, cells were collected and the periplasmic content was extracted. The receptor-binding domain of FimH was purified by dialysing it 4h at 4°C against 20 mM Na formate pH 4 and loading it on a Mono S HR column (Pharmacia Biotech). The protein was eluted with 20 mM Na formate, 1M NaCl pH 4. Fractions containing the FimH receptor-binding domain were pooled and dialyzed overnight at 4°C against 20 mM Hepes pH 8 and 150 mM NaCl, before crystallization.

### Co-crystallization of the FimH RBD with oligomannose-3

Crystallization conditions were screened at the high-throughput crystallization facility of the EMBL in Hamburg. Crystals grew at 292K, using the vapor diffusion method with sitting drops composed of 300 nL FimH-oligomannose-3 solution FimH at 13.6 mg/ml mixed with 2 mM oligomannose-3 in a 2∶1 molar ratio and 300 nL precipitant (Figure S1), equilibrated against a 100 µl reservoir of 1.0 M lithium sulfate, 0.1 M Tris-HCl at pH 8.5, 0.01 M nickel (II) chloride. To optimize these conditions, hanging drops were set up consisting of 1 µl FimH at 13.6 mg/ml mixed with 2 mM oligomannose-3 in a 2∶1 molar ratio and 1 µl of the precipitant solution, equilibrated against 500 µl of the same precipitant complemented with 3% glycerol. Glycerol has a beneficial effect on crystallization by preventing showering of the protein prior to nucleation, allowing a more controlled crystal nucleation and growth process.

### Structure determination and refinement

X-ray data have been collected to 2 Å resolution at the European Molecular Biology Laboratory (EMBL) beam line X12 at the Deutsches Elektronen Synchrotron (DESY, Hamburg, Germany). The crystal was flash cooled to 100K in the precipitant solution complemented with 30% isopropanol. All data were processed with DENZO and SCALEPACK from the *HKL* suite [62]. TRUNCATE from the CCP4 suite was used to calculate structure-factor amplitudes from the intensities. The structure has been solved by molecular replacement with MolRep [63] using the structure of the receptor-binding domain of FimH as the search model (PDB entry code 1UWF) [47]. The model was refined using rigid body refinement and the maximum likelihood function of CNS version 1.1 [64] and Refmac 5.2.0019 [65], with 5% of the data retained for cross-validation purposes. The initial molecular replacement solution was submitted to simulated annealing refinement. Successive positional and individual temperature factor refinements were alternated with manual model adjustment using TURBO-FRODO [66] and COOT [67] graphics.

### Structure analysis

A Ramachandran plot was drawn using Molprobity [68]. Baverage from the CCP4 suite [69] defined the average B-factors of the main chain atoms of the protein and of the water molecules. The packing contacts were analyzed using the CCP4 program CONTACT [69], using an intermolecular cut-off distance of 4 Å. Potential hydrogen bonds in the FimH-oligomannose-3 complex were identified using HBPLUS [70]. Subsequently, the predicted interactions were carefully checked with Coot [67]. Pictures have been generated using Pymol version 0.99.

### Bacterial binding in vitro to a human urothelial cell line

The *E. coli* strains AAEC185(pUT2002)(pMMB66) and AAEC185(pUT2002) have been used in bladder cell binding experiments. AAEC185 *E. coli* cells are *fim*-null mutants [72]. The pUT2002 plasmid carries the complete *fim* gene cluster with the deletion of *fimH*, resulting in FimH-deficient type 1 pili [72]. The *fimH* gene of J96 *E. coli* is located on the pMMB66 plasmid [53]. Strains AAEC185(pUT2002) and AAEC185(pUT2002)(pMMB66) were grown statically in 100 ml LB for 48 hours at 37°C to induce pili production. The bacterial cells were harvested under sterile condition by centrifugation at 3500 rpm for 20 min (Megafuse 1.0R, Heraeus instruments) and washed two times in phosphate buffered saline (PBS). The expression of type 1 pili was always checked using haemagglutination [26] prior to the infection of the bladder cells. Human bladder epithelial cell line 5637 (American Type Culture Collection HTB-9) was seeded in 12-well plates and cultured in Roswell Park Memorial Institute (RPMI) 1640 medium, supplemented with 10% fetal calf serum (FCS). The cells were maintained 2–4 days at 37°C in a humidified atmosphere containing 5% CO_2_ for confluent growth. The plate was washed 5 times with PBS complemented with 0.5 mM MgCl_2_ and 1 mM CaCl_2_ directly before use. Each well was incubated with 0.5 ml of 10^6^ to 10^7^ colony forming units (cfu) per ml in PBS. The plates were slowly shaken for 15 minutes to allow binding of the bacteria to the tissue cells. Five washes were performed with PBS to remove unbound bacteria. Bladder cells were lysed by adding 0.4 ml trypsin/EDTA for 10–15 minutes. Finally, the lysis was stopped by the addition of 10% FCS, when all cells were released from the plate. The input colony forming units (cfu) and the output cfu in each well were determined by 10-fold serial dilutions (1, 10^−2^, 10^−3^, 10^−4^, 10^−5^) in PBS and spotting 20 µl drops on LB-agar with the appropriate antibiotics. Inhibition of bladder cell binding was performed simultaneously as binding on the same 12-well plate, but with the bacterial inoculum pre-incubated with different concentrations of mannose or HM.

### In vivo bladder binding

All studies using mice were approved by the Animal Studies Committee of Washington University. Eight-week-old female C3H/HeN mice (NCI) were anesthetized and inoculated with a 50 µl suspension of ∼10^7^ UTI89 (in PBS or sugar solution) via transurethral catheterization [12]. Prior to inoculation, the inoculum was incubated for 20 min at 37°C with one of the following: 1 M methyl α-D-mannose, 0.5 mM heptyl α-D-mannose, 5 mM heptyl α-D-mannose. Six hours after inoculation, animals were euthanized, and their bladders harvested and homogenized in 1 mL of 0.025% Triton X-100/PBS. Bacterial titers were determined by plating serial dilutions of the homogenates on LB agar plates. Duplicate experiments of 5 mice each were performed.

### Gentamicin protection assay

UTI89 was grown overnight in LB and resuspended in PBS to an inoculum of ∼10^7^ cfu in 50 µl. The inoculum was then incubated for 20 min at 37°C with 5 mM methyl α-D-mannose, 0.5 mM heptyl α-D-mannose, 5 mM heptyl α-D-mannose, or PBS. After incubation, 6–7 week old C3H/HeN mice were inoculated via transurethral catheterization [12]. An *ex vivo* gentamicin protection assay was performed as previously described [11]. Briefly, at 1 hour post-infection, the mice were sacrificed and bladders were dissected aseptically. Each bladder was washed 3 times with sterile PBS. The washes were collected and plated to obtain the luminal fraction of bacteria. The bladders were then treated with 100 µg/ml gentamicin for 90 min at 37°C. After treatment, the supernatant was removed and titered to ensure efficient killing of extracellular bacteria. The bladders were washed twice more to remove residual gentamicin and homogenized in 1 mL 0.025% triton X-100/PBS and bacterial counts were determined by plating serial dilutions on LB agar plates.

### Biofilm assay

UTI89 was grown overnight in LB broth at 37°C with shaking and diluted 1∶1000 in LB or LB with varying amounts of methyl α-D-mannose or heptyl α-D-mannose. 96-well round bottom polyvinyl chloride plates (Falcon) were sterilized in tissue culture hood under UV irradiation for at least 30 minutes. 100 µl of the solutions were then added to the sterile PVC plate, 6 wells per variable. LB without bacteria was added to 6 wells as a blank. The plate was incubated for 48 hours at room temperature, washed 3 times in PBS and allowed to dry. 125 µl of 1% crystal violet solution was added to each well for 10 minutes. After staining, the plates were washed again in PBS 3 times and allowed to dry. The crystal violet was solubilized with 150 µl of 33% acetic acid, 100 µl was transferred to a flat bottom plate and absorbance was read at 600 nm.

### Protein Data Bank accession number

The coordinates and the structure factors have been submitted to the Protein Data Bank with accession codes 2vco and r2vcosf respectively.

## Supporting Information

Figure S1 Crystals of the FimH receptor-binding domain in complex with oligomannose-3. The crystals were grown by the vapour diffusion method in 1.0 M Li_2_SO_4_, 0.1 M Tris pH 8.5, 0.01 M NiCl_2_, A, in sitting drop, diffracting to 2.6 {Angstrom} resolution, and B, in hanging drop, optimized by the addition of 3% glycerol to the precipitant and diffracting to a maximum resolution of 2.0 {Angstrom}.
(5.22 MB TIF)

Table S1 Data collection and processing, refinement statistics and model quality.
(0.02 MB DOC)
