# Correction: Intervening with Urinary Tract Infections Using Anti-Adhesives Based on the Crystal Structure of the FimH–Oligomannose-3 Complex

**DOI:** 10.1371/annotation/ea59d179-0a71-4836-86f0-8d375f5df089

**Published:** 2008-06-09

**Authors:** Adinda Wellens, Corinne Garofalo, Hien Nguyen, Nani Van Gerven, Rikard Slättegård, Jean-Pierre Hernalsteens, Lode Wyns, Stefan Oscarson, Henri De Greve, Scott Hultgren, Julie Bouckaert

Affiliation 1 is incorrect. It should read: Department of Molecular and Cellular Interactions, VIB, Brussels, Belgium

