# Supplementary figures and images for: Intervening with Urinary Tract Infections Using Anti-Adhesives Based on the Crystal Structure of the FimH–Oligomannose-3 Complex
Source: PLoS One. 2008 Apr 30;3(4):e2040. doi: 10.1371/journal.pone.0002040 (PMC2323111; doi:10.1371/journal.pone.0002040)

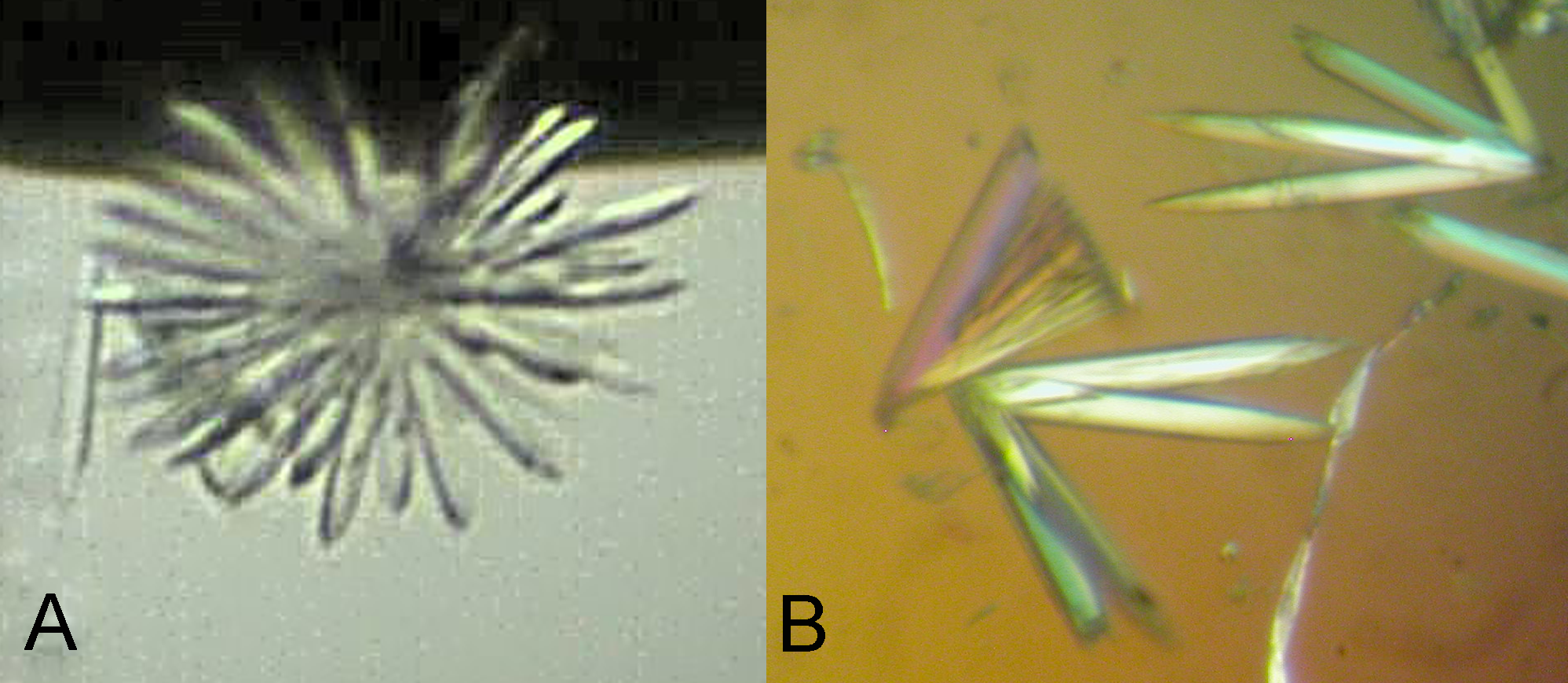

Supplement: Figure S1 — Crystals of the FimH receptor-binding domain in complex with oligomannose-3. The crystals were grown by the vapour diffusion method in 1.0 M Li2SO4, 0.1 M Tris pH 8.5, 0.01 M NiCl2, A, in sitting drop, diffracting to 2.6 {Angstrom} resolution, and B, in hanging drop, optimized by the addition of 3% glycerol to the precipitant and diffracting to a maximum resolution of 2.0 {Angstrom}. (5.22 MB TIF) [file pone.0002040.s001.tif]
