# Supplementary material for: Intervening with Urinary Tract Infections Using Anti-Adhesives Based on the Crystal Structure of the FimH–Oligomannose-3 Complex
Source: PLoS One. 2008 Apr 30;3(4):e2040. doi: 10.1371/journal.pone.0002040 (PMC2323111; doi:10.1371/journal.pone.0002040)
Supplement: Table S1 — Data collection and processing, refinement statistics and model quality. (0.02 MB DOC) [file pone.0002040.s002.doc]

**Table S1.** **Data collection and processing, refinement statistics and model quality**

Space group P3_1_21

Unit cell dimensions (Å) a = b = 90.562 c = 79.480

Molecules per asymmetric unit 2

Solvent content (%) 55.6

Resolution range (Å) 78.33 – 2.1

Reflections (total/unique) 142,687 / 22,429

Completeness (%) ^a^ 99.8 (100.0)

R_merge_ ^a,b^  0.099 (0.42)

<I/σ(I)> ^a,b^ 16.0 (5.8)

R_free_ 0.241 (0.310)

R_cryst_ 0.184 (0.228)

R_all_ 0.187

Ramachandran

% of residues in favored regions 97.12

allowed regions 100.00

Rmsd bond lengths (Å) 0.010

Rmsd bond angles (º) 1.178

Average B-factors main chain 24.4

Average B-factors water 36.1

^a^ Values in parentheses indicate statistics for the highest resolution shell (2.14 - 2.1 Å). ^b^ *R*_merge_ = Σ**_h_** Σ*_i_* |I**_h_**,*_i_* - <I**_h_**>|/ Σ**_h_** Σ*_i_* <I**_h_***>*, where I**_h_***_,i_* is the *i*th observation of reflection **h** and <*I***_h_**> is the weighted average intensity for all observations *i* of reflection **h***.*
